# A novel type of cosavirus from children with nonpolio acute flaccid paralysis

**DOI:** 10.1186/s12985-016-0630-3

**Published:** 2016-10-12

**Authors:** Yan Yang, Aiping Ju, Xiaofen Xu, Xinyu Cao, Ying Tao

**Affiliations:** 1 The Fourth Affiliated Hospital of Jiangsu University, 20 Zhengdong Road, Zhenjiang, Jiangsu 212001 China; 2 Department of Clinical Laboratory, Women and Children’s Hospital of Huadu District, Guangzhou, Guangdong 510800 China

**Keywords:** Human cosavirus, Complete genome, Phylogenetic analysis

## Abstract

Human cosavirus (HCoSV) is a genus recently identified in the family Picornaviridae, which contains important pathogens to human health. Here, a novel type of HCoSV strain, cosavirus-zj-1 (GenBank no. KX545380), was identified in the fecal sample of a child with nonpolio acute flaccid paralysis (AFP) in China. Phylogenetic and sequence analyses suggested that this virus strain belonged to a new genotype in HCoSV B species. Our data show that surveillance of HCoSV is necessary for detecting viral agents in children with AFP, despite being the low detection rate.

## Findings

Human Cosavirus (HCoSV) is a new member in the *Picornaviridae* family. It was originally detected from fecal samples of both healthy children and non-polio acute flaccid paralysis (AFP) patients in Pakistan and Afghanistan as well as in a fecal sample of a 64-year- old woman from Scotland [1, 2]. Subsequently, many studies on HCoSV detection in children and adults in different countries were reported [3–9].

Besides the apparently wide geographic distribution, HCoSV has a wide genetic diversity. The genome of HCoSV is about 7.6 kb long organized in a typical picornavirus genome, the only difference is the absence of the leader (L) sequence [1]. The genome encodes four structural viral proteins (VP4, VP2, VP3, and VP1), and nine nonstructural proteins (2A1, 2A2, 2B, 2C, 3A, 3B1, 3B2, 3C, and 3D) [1]. Based on the VP1 sequences, the genus is currently divided into six genetically distinct species (A-F) including more than 30 genotypes [10]. Similar to cardioviruses, which are close relatives of picornaviruses, HCoSV was frequently detected in the feces of symptomatic as well as asymptomatic subjects, and thus their role in human enteric disease remains unclear. This is partly because there have been only a limited number of epidemiological studies on this emerging virus.

In the present study, a total of 29 fecal samples were collected from 29 children between January 2010 and December 2014 with clinical manifestations of AFP but no detection of poliovirus. RT-PCR method was used to amplify a 316-nucleotides fragment of the 5′ non-coding region of the HCoSV genome [1]. Of the 29 fecal samples investigated, only one was positive for HCoSV. The PCR products were T-A cloned, sequenced and then compared to the NCBI nucleotide collection using BLASTn. This revealed that the 316 nt HCoSV fragment shared highest sequence identity (86 %) with a viral sequence belonging to HCoSV A species (HCoSV-A1, FJ438902). In order to get the full genome of this divergent HCoSV strain and investigate whether this fecal sample also contain other viral pathogens, viral metagenomic method was used to detect the viral nucleic acid in this sample as previously reported [11].

The viral metagenomic sequencing of the HCoSV-positive fecal sample produced a total of 810,928 raw sequence reads using the Illumina MiSeq platform (2 × 250 cycles), of which 432 were identified as HCoSV by BLASTx search. The 432 HCoSV sequence reads were *de novo* assembled using Geneious 8.0 resulting in three contigs covering 75.2 % (5369/7132) of the complete genome of HCoSV. Also identified in the metagenomic dataset were 24 anellovirus reads. The anelloviruses are endemic worldwide and present in many different tissues [12], but there is, as of yet, no proof that anelloviruses cause any disease. Therefore, the anelloviruses in the fecal samples was not considered to be the causative agents of the APF in the present study. The near complete genome of the HCoSV was then obtained by PCR to bridge sequence gaps based on the three HCoSV sequence contigs assembled from viral metageomic data.

The nearly complete genome of HCoSV identified here was 7132 bp long and has been tentatively named cosavirus-zj-1 with a GenBank accession number of KX545380. A pairwise comparison of the near-genome sequence showed that cosavirus-zj-1 had the highest nucleotide similarity (67.8 %) with an HCoSV B strain (FJ438907) that was identified from children with AFP in Pakistan [1]. This HCoSV encodes a 2131 aa polyprotein. Sequence analysis indicated the polyprotein, P1 protein, and 2C +3CD protein of cosavirus-zj-1 shared the highest sequence identities of 69.0, 73.9, and 68.3 %, respectively, with the closest relative (FJ438907) in GenBank. The ICTV states that enteroviruses sharing >70 % amino acid identity in polyprotein, >60 % amino acid identity in P1 protein, and >70 % amino acid identity in 2C +3CD regions belong to the same species. Based on the criteria, the cosavirus strain identified in the present study likely belongs to a new genotype within HCoSV B species. A numerous enterovirus serotypes have been genetically characterized based on VP1 sequence and variants showing greater than 88 % amino acid identity in their VP1 have been shown to belong to the same antibody neutralization serotype [13]. Although the VP1 of cosavirus-zj-1 shared amino acid sequence identity of 70.2 % with its closest relative (FJ438907), whether this cosavirus strain belonged to a new serotype of HCoSV B species needs further serologic study.

**Fig. 1:**
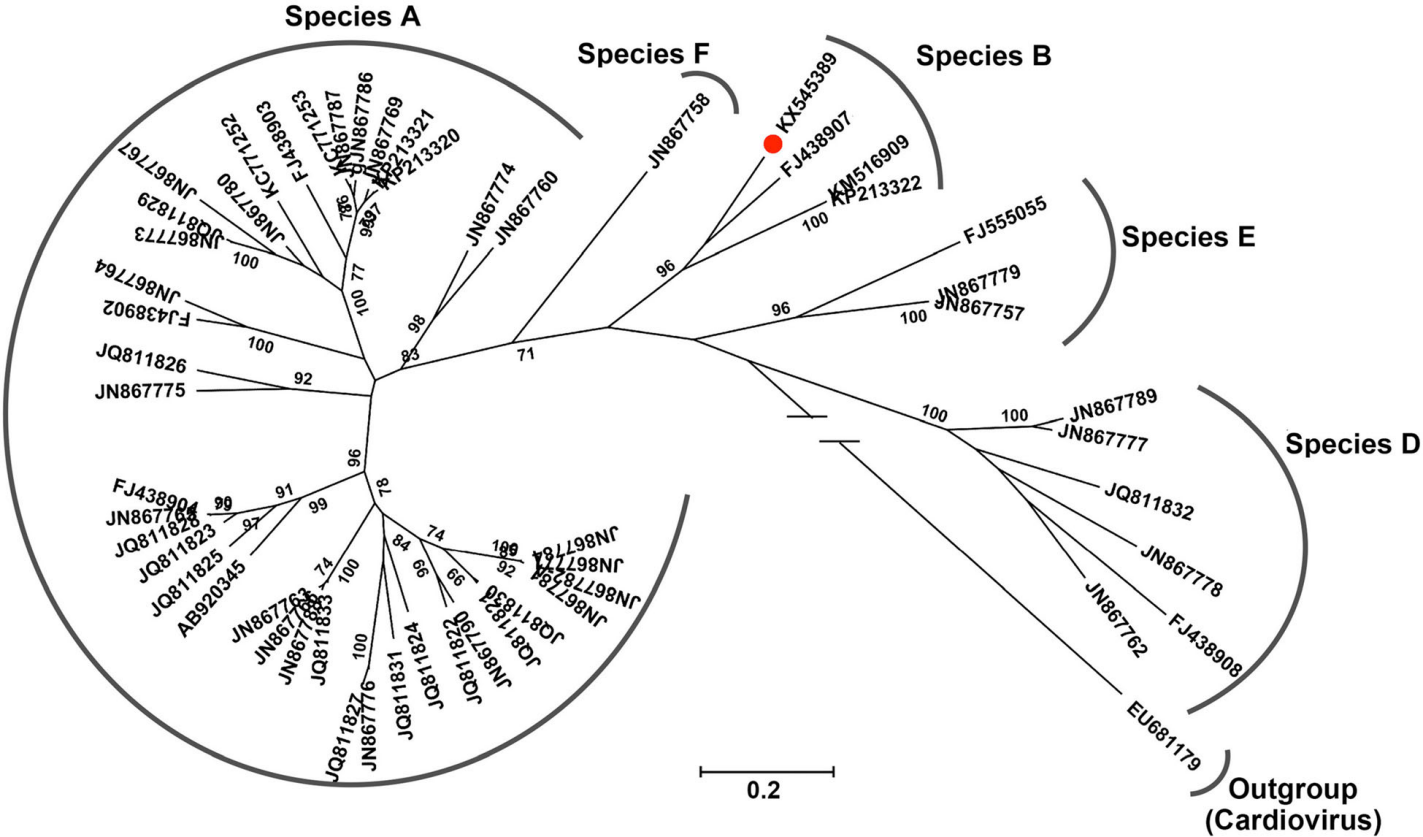
Phylogenetic tree based on the amino acid sequence of VP1 depicting relationships among the members of the genus of Human cosavirus. The newly discovered cosavirus-zj-1 is indicated by a *black dot*

To determine the relationship between cosavirus-zj-1 in the present study and the other members of HCoSVs, phylogenetic analysis based on the VP1 amino acid sequences was performed. All of HCoSV strains with complete or nearly complete VP1 region available in GenBank were included in the phylogenetic analysis. Amino acid sequences were aligned by CLUSTALW, and phylogenetic trees were constructed using MEGA 5.0 software in Maximum likelihood method mode with 500 bootstrap replicates. Results showed that cosavirus-zj-1 clustered with other three HCoSV strains, including the previous HCoSV species B strain (FJ438907), and two unpublished HCoSV sequences, KP213322 and KM516909, which were detected from feces from children in China according to the sequence annotation in GenBank (Fig. 1). The P1 protein sequence of KP213322 shared >60 % identity with HCoSV species B strain (FJ438907) and cosavirus-zj-1 in this study, we therefore also grouped them together into species B (Fig. 1).

## Conclusion

Taken together, we identified a cosavirus belonging to a new genotype of HCoSV species B from feces of a child with AFP in China. The cosaviral sequence in the fecal sample also confirmed by viral metagenomics which indicated that although this sample was also positive for a small number of anellovirus, cosavirus might be the cause of the AFP of this child. It is the first report of HCoSV species B strain was detected in a patient with nonpolio AFP in China, which suggested that surveillance of HCoSV is important for detecting viral agents in children with AFP, despite being the low detection rate.

## Abbreviations

AFP: Acute flaccid paralysis
HCoSV: Human cosavirus
PCR: Polymerase chain reaction
